# Five families of diverse DNA viruses comprehensively restructure the nucleus

**DOI:** 10.1371/journal.pbio.3002347

**Published:** 2023-11-06

**Authors:** Quincy Rosemarie, Bill Sugden

**Affiliations:** Department of Oncology, McArdle Laboratory for Cancer Research, School of Medicine and Public Health, University of Wisconsin-Madison, Madison, Wisconsin, United States of America

## Abstract

Many viruses have evolved ways to restructure their host cell’s nucleus profoundly and unexpectedly upon infection. In particular, DNA viruses that need to commandeer their host’s cellular synthetic functions to produce their progeny can induce the condensation and margination of host chromatin during productive infection, a phenomenon known as virus-induced reorganization of cellular chromatin (ROCC). These ROCC-inducing DNA viruses belong to 5 families (herpesviruses, baculoviruses, adenoviruses, parvoviruses, and geminiviruses) that infect a wide range of hosts and are important for human and ecosystem health, as well as for biotechnology. Although the study of virus-induced ROCC is in its infancy, investigations are already raising important questions, such as why only some DNA viruses that replicate their genomes in the nucleus elicit ROCC. Studying the shared and distinct properties of ROCC-inducing viruses will provide valuable insights into viral reorganization of host chromatin that could have implications for future therapies that target the viral life cycle.

## Abstract 2

Many viruses have evolved ways to restructure their host cell’s nucleus profoundly and unexpectedly upon infection. This Essay looks at the phenomenon of cellular chromatin reorganization and asks what we can learn from studying the five diverse families of viruses that induce it.

## Introduction

Life, as we currently know it on Earth, is defined as being cellular and DNA based. Viruses, although nucleic acid based, are not cellular and can be thought of as cellular parasites that encode information allowing them to replicate, spread, escape, and infect additional hosts. Of the 3 domains of our biological tree of life (Archaea, Bacteria, and Eukarya) [1], only Eukarya store their DNA in a subcellular compartment, the nucleus. In a normal resting eukaryotic cell, meters of DNA are wrapped around histone octamers or nucleosomes [2] that reside within the nucleus. Arrays of these nucleosomes constitute the chromatin fibers [3] that form chromosomes, which are spatially localized to distinct regions of the nucleus [4]. Although the cell’s chromatin typically fills much of the nuclear volume, specific cellular events can lead to its reorganization.

**Fig 1:**
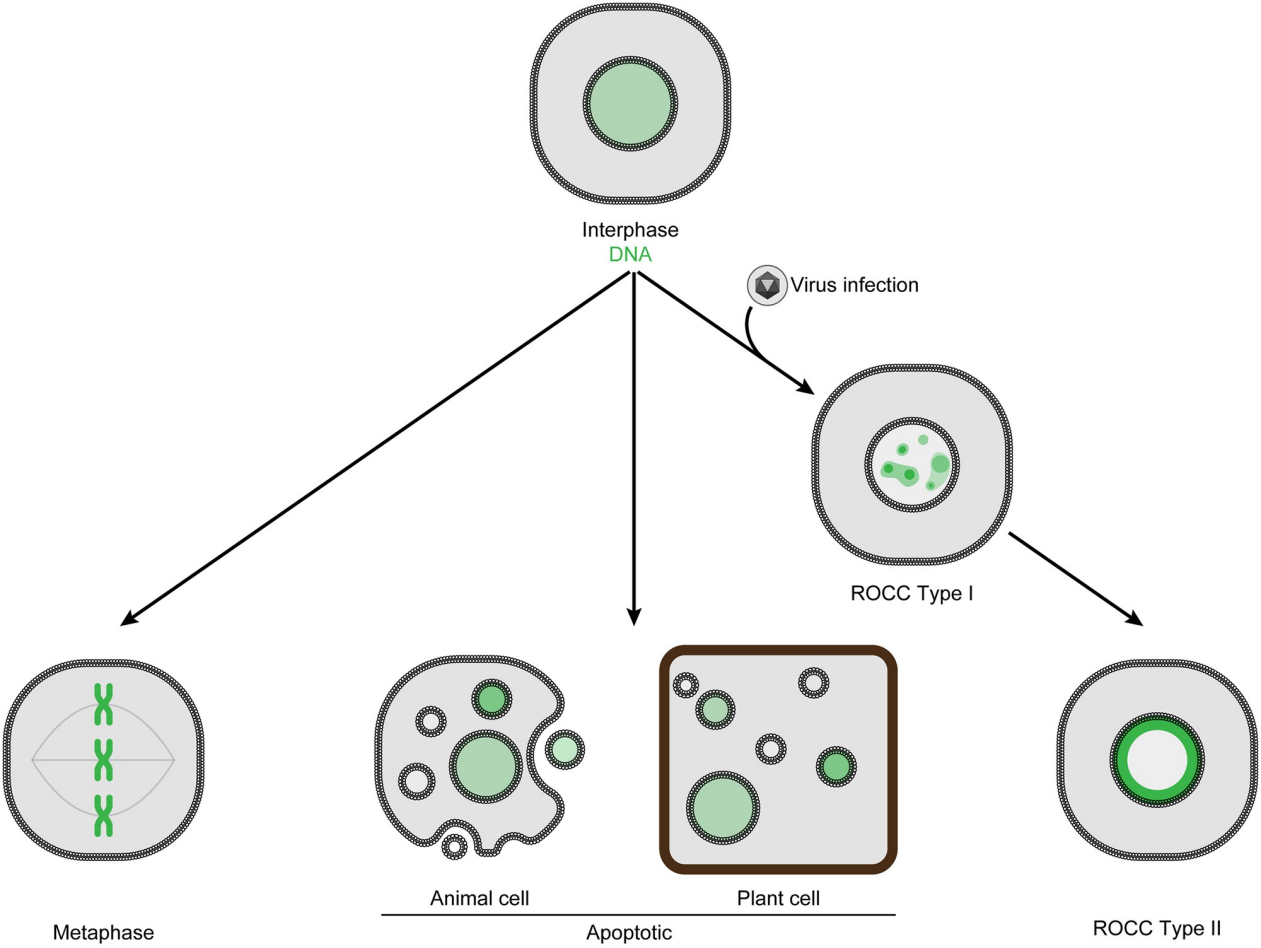
Forms of cellular chromatin reorganization. In a normal, resting, interphase, eukaryotic cell, cellular DNA resides in the nucleus as uncondensed, protein-associated chromatin. Cellular chromatin is normally distributed evenly across the nucleus, but specific cellular events can trigger its reorganization. For example, during cell division, chromatin is condensed into X-shaped chromosomes, prominently seen in metaphase cells (left). When a cell undergoes apoptosis (middle), its chromatin is condensed and eventually becomes fragmented, often budded out through plasma membrane blebbing. Upon infection with some viruses, virus-induced reorganization of cellular chromatin (ROCC) occurs (right). First, cellular chromatin becomes condensed (ROCC type I), then, as viral DNA replication and productive infection progress, the condensed chromatin becomes marginated along the periphery of the nucleus (ROCC type II).

One common form of chromatin reorganization occurs during cell division, when cellular chromatin is condensed into X-shaped chromosomes (Fig 1). As the nuclear envelope breaks down at the beginning of mitosis and meiosis, these condensed metaphase chromosomes are arranged across the cellular equator in preparation for their separation into 2 daughter cells. A second form of chromatin reorganization occurs during apoptosis. In animal cells, pyknosis (the irreversible condensation of chromatin within the nucleus) and karyorrhexis (the fragmentation of the cell nucleus and its condensed chromatin) occur, causing the uneven distribution of apoptotic bodies containing DNA throughout the cytoplasm [5]. In plants, apoptosis-like events lead to condensed chromatin and vacuolar cell death, in which apoptotic bodies are also observed [6]. A third kind of chromatin reorganization is induced by some viral infections and is characterized by the condensation and margination of cellular chromatin within the cell nucleus. This reorganization has been previously referred to as virus-induced reorganization of cellular chromatin (ROCC) [7] and likely occurs in 2 stages (ROCC type I and ROCC type II; Fig 1).

Of the different organized states of cellular chromatin, virus-induced ROCC remains the least understood and is the focus of this Essay. Here, we consider the insights that can be gained from considering the properties of the viruses that induce ROCC and examine the factors known to contribute to virus-induced ROCC and the potential mechanisms that could mediate it. Given the ubiquity of virus-induced ROCC, a greater understanding will likely lead to further insights into how the viruses that induce it manipulate their hosts, and how to intervene therapeutically in their life cycles.

## What does virus-induced ROCC look like?

**Fig 2:**
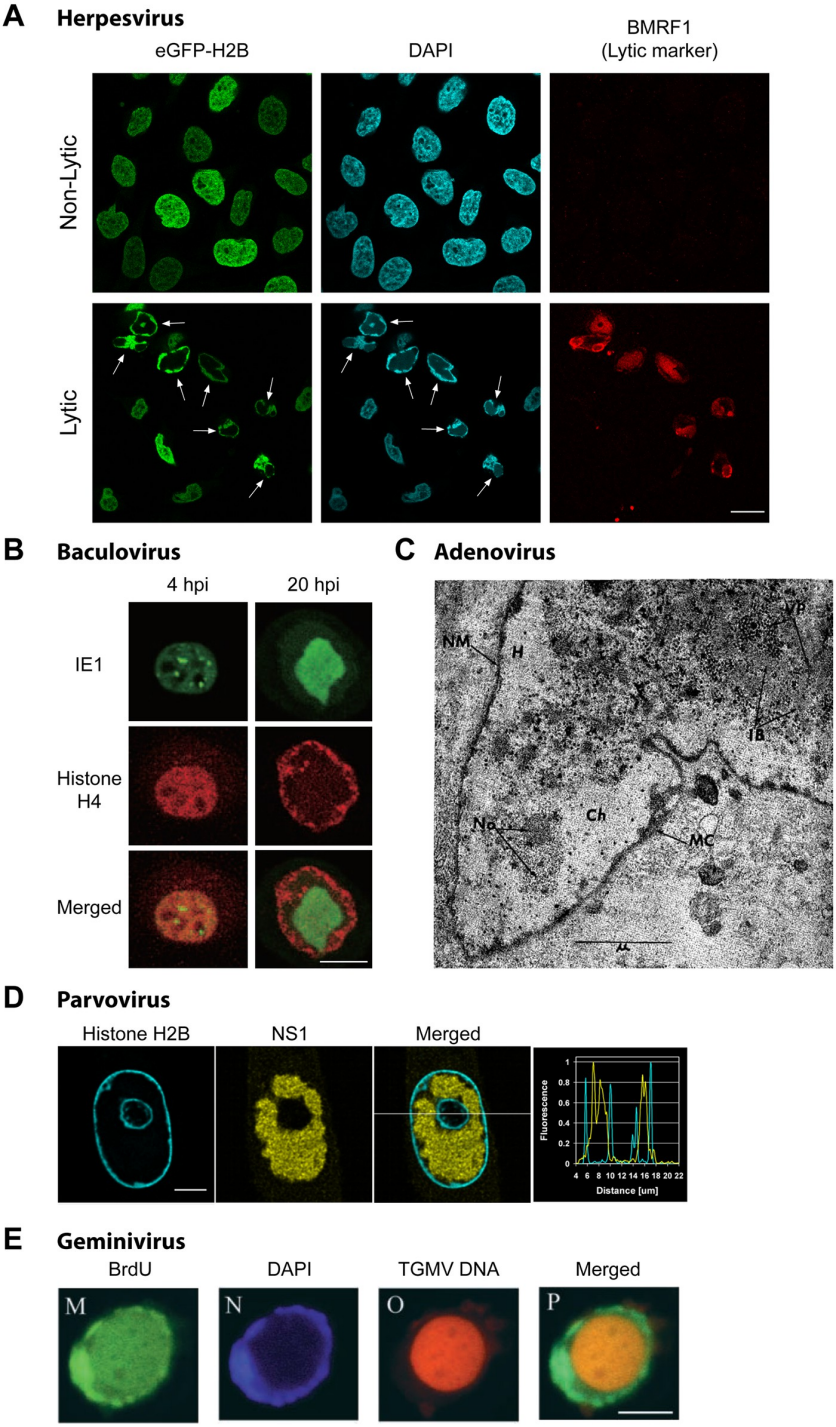
Virus-induced chromatin reorganization in infected cells. Representative images of chromatin reorganization upon **(A)** the lytic infection of herpesvirus (EBV) and infections with **(B)** baculovirus (BmNPV) [8], **(C)** adenovirus (canine hepatitis virus; CAV-1) [9], **(D)** parvovirus (CPV) [10], and **(E)** geminivirus (TGMV) [11]. Images are adapted from their respective publications as indicated. Cellular DNA (fluorescent-tagged histones, or DAPI staining) and viral DNA (TGMV DNA probe) or viral proteins indicative of its replicative compartments (BMRF1, IE1, NS1) were visualized as indicated. **(A, B, D, E)** Fluorescence microscopy images of infected cells and **(C)** an electron micrograph of an infected cell. Virus-induced ROCC can be observed for each example of infected cells, characterized by the condensation and margination of cellular chromatin within the cell nucleus. BMRF1, an early gene of EBV; IE1, baculovirus immediate early protein 1; NS1, parvovirus nonstructural protein 1. In **(C)**: MC, marginated chromatin; Ch, chromatin, VP, viral particles; H, halo; IB, inclusion body; NM, nuclear membrane; No, nucleolus. Scale bars: **(A)** 20 μm, **(B and E)** 10 μm, **(C)** 1 μm, **(D)** 5 μm. BmNPV, *Bombyx mori* nucleopolyhedrovirus; CAV-1, canine adenovirus type 1; CPV, canine parvovirus; EBV, Epstein–Barr virus; ROCC, reorganization of cellular chromatin; TGMV, tomato golden mosaic virus.

Five widespread, successful families of DNA viruses (herpesviruses, baculoviruses, adenoviruses, parvoviruses, and geminiviruses) share the surprising function of inducing ROCC. Members of these families reorganize cellular chromatin during their productive infections (the time when they produce progeny virus), causing cellular DNA to concentrate at the periphery of the nucleus (Fig 2).

Virus-induced ROCC was first noted about 75 years ago, and since then, ROCC has been observed for multiple viruses [12]. Early studies of herpesvirus infections in embryonated eggs revealed alterations in the nuclei of infected cells and increased concentrations of DNA at the periphery of the nuclei [12]. Later, electron microscopy and fluorescence microscopy studies in herpesvirus-infected mammalian cells confirmed the finding of marginated chromatin in productively infected cells [13–16], consistent with ROCC. Studies of adenovirus infections revealed similar phenomena: Electron microscopy assays of adenovirus infections showed that the virus induces a margination of DNA in infected nuclei [9], and fluorescence in situ hybridization (FISH) localized the adenoviral DNA to the center of infected nuclei, with the cellular DNA at the nuclear periphery [17]. Parvovirus infection of mammalian cells with fluorescently tagged histones also induced a similar condensation of the labeled chromatin to the nuclear rim [10]. Parallel approaches with geminiviruses demonstrated a similar disposition of viral and cellular DNAs in infected plant cells [11,18,19]. And, baculoviruses, a virus family that infects a wide range of insects, similarly reorganize cellular chromatin [8,20].

Early examples of virus-induced chromatin reorganization were found through electron microscopy or immunofluorescence microscopy of fixed cells. More recently, observations of ROCC in action have been made possible by the development of methods that allow the detection of viral DNA in live cells. Polymers of bacterial DNA elements, such as *parS* from some *Burkholderiales* species or the *lac* operon from *Escherichia coli*, have been integrated into the viral DNA and detected upon binding specific fluorescent proteins. *parS* has been used to visualize ROCC in live cells infected productively by adenoviruses and human cytomegalovirus (HCMV) [21,22]. The *lac* operon, on binding its repressor fused to mCherry, has been used to follow the genomes of Epstein–Barr virus (EBV) in latently infected cells induced to support the viral lytic (that is, productive) cycle [23]. These viral genomes were first amplified when the cells entered the beginning of S-phase, and the cellular chromatin was progressively compacted and marginated as compartments containing the amplifying viral DNA expanded.

**Fig 3:**
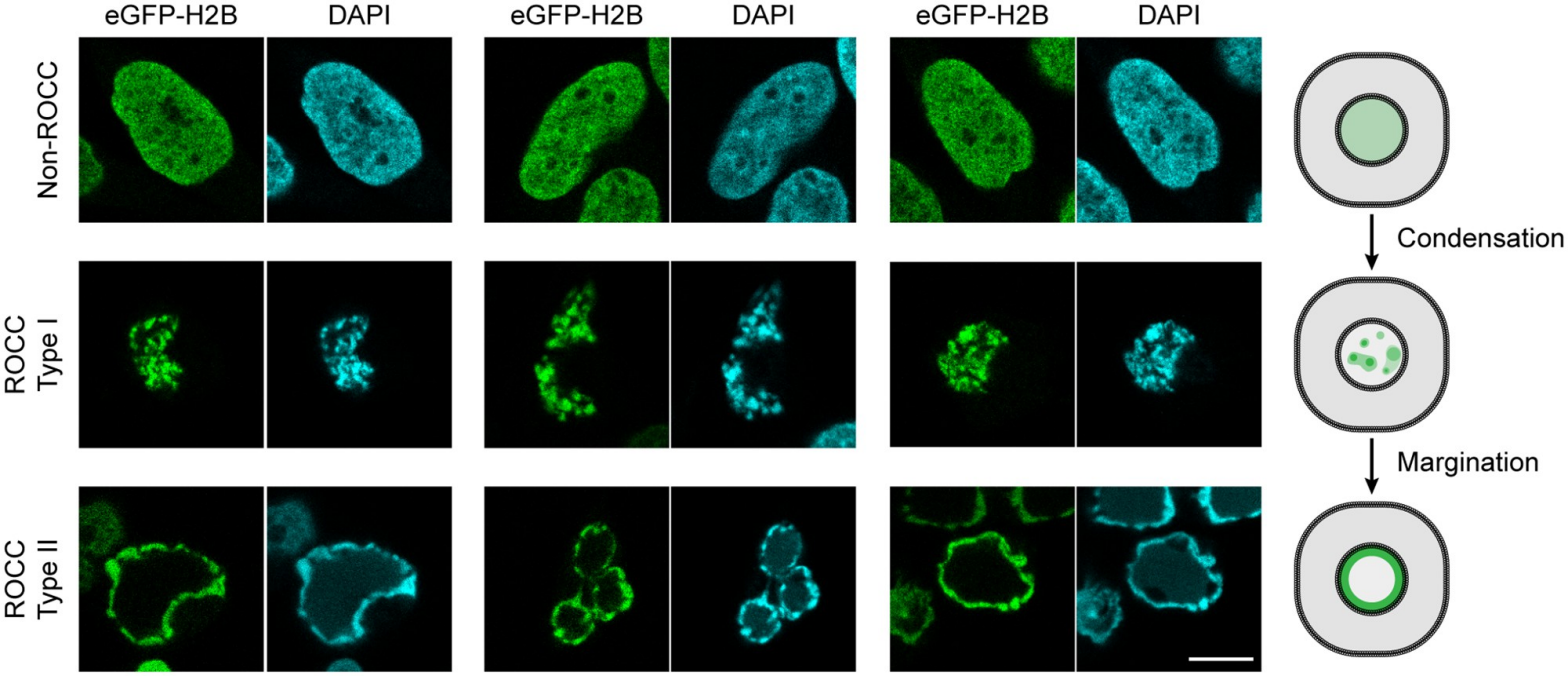
Two types of chromatin reorganization in EBV lytic cells. The progression of reorganizing chromatin in EBV lytic cells can be described in 2 steps: ROCC type I, characterized by host chromatin condensation without margination; and ROCC type II, defined by both the condensation and margination of cellular chromatin to the periphery of the nucleus. ROCC type I is triggered upon the formation of a viral DNA synthesis complex and its initiation of synthesis. The extensive DNA synthesis that follows gives rise to ROCC type II. The cell line used in this figure is the EBV-positive cell line, iD98/HR1 [23]. Type I reorganization is detected by treating the lytic-induced cells with viral DNA synthesis inhibitors such as GCV or PAA. Close examination of the DAPI channel of cells supporting type II reorganization reveals DAPI signals of lower levels within the “void” of the marginated chromatin. These signals come from the newly synthesized viral genomes. Accordingly, these “void” regions have only background levels of eGFP-H2B signals, indicating the histone-free state of these viral DNA molecules. Scale bar: 10 μm. EBV, Epstein–Barr virus; GCV, ganciclovir; PAA, phosphonoacetic acid; ROCC, reorganization of cellular chromatin.

A specific phenotype of virus-induced ROCC is observed in 2 of the families but is likely to be generally required by all 5 virus families. Both geminiviruses and herpesviruses seem to condense cellular DNA prior to margination [7,19]. Geminiviral infection of tobacco cells leads to extensive condensation of cellular DNA, rendering it akin to “prophase-like fibers,” which are likely precursors to the compaction of host chromatin and its movement to the nuclear periphery [19]. Similarly, blocking extensive viral DNA synthesis in EBV-infected cells induced to enter their productive phase supports the condensation of cellular DNA, but not its margination [7]. It thus seems likely that the condensation of cellular chromatin precedes its margination. Accordingly, the condensation of chromatin without its margination is termed ROCC type I, and the condensed and marginated cellular chromatin is termed ROCC type II (Fig 3). To understand ROCC better, it will be important to test if both ROCC type I and II are shared by all ROCC-inducing virus families.

## What can we learn from ROCC-inducing viruses?

**Fig 4:**
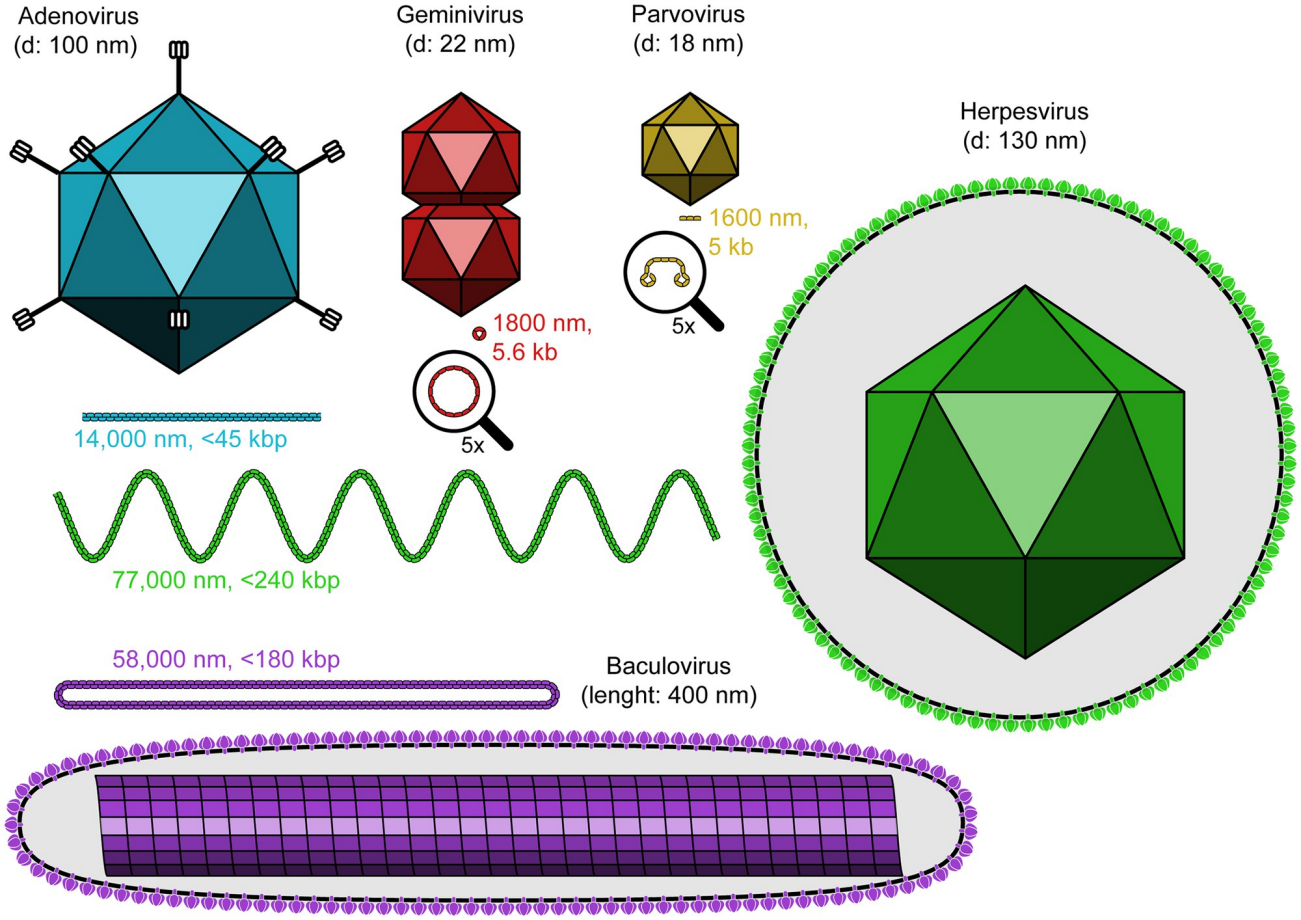
Physical properties of the 5 ROCC-inducing virus families. The virus particles and their DNA genomes of the ROCC-inducing families are depicted with their particles and genomes separately to scale. While the diameters of the viral icosahedrons vary approximately 7-fold [24–29], the lengths of their corresponding DNAs vary approximately 100-fold [30–34]. The genomic DNAs of parvoviruses and geminiviruses are single-stranded; those of the 3 other families are double-stranded. Baculoviral and geminiviral DNAs are circular; the others are linear molecules. Baculoviruses have rod-shaped capsids encapsidating their DNAs, while the others are icosahedral in shape, with adenoviruses having fibers that protrude from the vertices [29,33]. Geminiviruses have “twin particles,” hence their name “gemini,” meaning twins [27]. Both baculoviruses and herpesviruses have particles that are enveloped, that is, surrounded by lipid-containing membranes, while the other 3 families are nonenveloped. These envelopes are studded with multiple, distinct, virally encoded glycoproteins. d, capsid diameter (in nm), measured across opposing vertices. Genome length is given in nm (measured as linear molecules) and kilobase/kilobasepair (kb/kbp).

The 5 families of ROCC-inducing viruses infect plants, invertebrates, and vertebrates. Although these virus families are diverse in their hosts, sizes, and structures (Fig 4), they also share several significant properties (Table 1). Comparing and contrasting the properties of these viruses provides important insights into the surprisingly common phenomenon of virus-induced ROCC, including how these properties contribute to chromatin reorganization and what mediates it.

**Table 1:** Properties of 5 families of viruses that support reorganization of chromatin.

	Herpesviruses (*Herpesviridae*)	Baculoviruses (*Baculoviridae*)	Adenoviruses (*Adenoviridae*)	Parvoviruses (*Parvoviridae*)	Geminiviruses (*Geminiviridae*)
Host	Vertebrates	Insects	Vertebrates	Mammals, arthropods, reptiles	Plants, insects
Envelope	Enveloped	Enveloped	Nonenveloped	Nonenveloped	Nonenveloped
Capsid structure	Icosahedral (T = 16) [24,25]	Rod-shaped [33,35]	Icosahedral (T = 25) [26,29,36]	Icosahedral (T = 1) [28,37,38]	Twinned icosahedral (T = 1) [27]
Length of virion DNA	125–240 kbp	80–180 kbp	26–45 kbp	5 kb	2.7–5.2 kb
Virion nucleic acid	Linear dsDNA	Circular dsDNA [33]	Linear dsDNA	Linear ssDNA	Circular ssDNA [39]
**Viral template for early transcription** ^a^	**dsDNA, chromatinized** [40,41]	**dsDNA, chromatinized** [42]	**dsDNA, chromatinized** [43]	**dsDNA, chromatinized** [44,45]	**dsDNA, chromatinized** [46–48]
**Histone regulation of early transcription** ^a^	**Yes**	**Yes**	**Yes**	**Yes**	**Yes**
Viral DNA polymerase	Yes [49,50]	Yes [33]	Yes [32]	No [37,45]	No [51]
Simultaneous cellular DNA synthesis	No [23,52,53]	No [33]	No [54]	Yes/No [55–58]	Yes [11,51]
**Site of genome packaging** ^a^	**Nucleus**	**Nucleus**	**Nucleus**	**Nucleus**	**Nucleus**
**Cellular histone coating packaged virion DNA** ^a^	**No** [52,59,60]	**No** [35,61,62]	**No** [63]	**No**	**No**
Coating on encapsidated genome	Spermine, spermidine (putative) [59]	Viral P6.9 [35,61,62]	Viral protein VII [63]	Unknown	Unknown

^a^Properties shared across all 5 families of viruses.

### General mechanistic insights

The first insight comes from the genomic properties of these virus families. All 5 families are DNA viruses, although the form of their DNA differs, being either single-stranded or double-stranded when packaged. Their genome sizes cover a broad range, too: from 2.7 kb for geminiviruses up to 240 kbps for members of the baculovirus and herpesvirus families (Fig 4). This approximately 100-fold span of genome lengths provides an insight into their shared function of reorganizing chromatin. The small genomes of geminiviruses and parvoviruses encode so few genes that it is unlikely that they directly mediate this reorganization. Rather, we need to seek a mechanism that carries it out, at least in part, using host machinery.

A second insight can be derived from the nature of the viral and cellular DNA synthesis in infected cells. In contrast to the almost universal dependence of ROCC-inducing virus families on their hosts’ RNA polymerases for transcription, the families of the 3 larger viruses (herpesviruses, baculoviruses, and adenoviruses) encode their own DNA polymerases [32,33,49]. Parvoviruses and geminiviruses, on the other hand, rely on the DNA replication machinery of their hosts [34,45,51]. A potential corollary can be found in the effects of the replication of these viruses on their host DNA synthesis. Herpesviruses, baculoviruses, and adenoviruses inhibit cellular DNA synthesis, whether through the induction of cell cycle arrest or the inhibition of histone synthesis and/or cellular DNA synthesis itself [23,52–54,64,65]. Parvoviruses can synthesize their DNA either in the absence of cellular DNA synthesis or in its presence, depending on which cellular DNA polymerases they use. For example, human bocavirus 1 (HBoV1) can replicate in differentiated, nondividing cells using the DNA repair polymerases Pol η and Pol κ in the absence of cellular DNA synthesis [55]. By contrast, minute virus of mice (MVM) relies primarily on Pol α and Pol δ (enzymes used during cellular synthesis) for its own amplification in cells that are synthesizing their own DNA (personal communication, Dr. Kinjal Majumder). Geminiviruses can induce host DNA synthesis in cells in which they replicate their DNA and also use Pol α and δ for their synthesis [51]. This varied association with chromosomal synthesis suggests that inhibition of cellular DNA synthesis is not essential for virus-induced ROCC.

The third insight into virus-induced chromatin reorganization can be gained from the observation that all 5 virus families package their genomes into their capsids within the host nucleus, and those packaged genomes are free of cellular histones [59,61–63,66]. This absence contrasts with the fact that they all use histone-bound templates for transcribing their RNA, at least at an early stage during their productive infections. Some of these viruses encode their own histone-like proteins that bind to their DNA. For example, the baculovirus protein P6.9 is a protamine-like protein that coats encapsidated genomes and is essential for the production of infectious virus [35,61,62]. Adenoviruses also encode a histone-like protein, protein VII, which is not required for viral genomes to be packaged [63] but binds host chromatin to modify its composition [67]. Encapsidated herpesvirus genomes are also thought to be coated with polyamines [59]. Ultimately, for all 5 families, the viral genomes packaged in their virions are histone free. This absence of cellular histones in virus particles suggests that the viruses that induce ROCC need to separate their own histone-free, newly synthesized genomes, which will be encapsidated, from the histone-bound cellular chromatin.

### Which viral genes are involved in ROCC?

Studies with baculoviruses and herpesviruses have identified viral genes required for the reorganization of chromatin. The transfection of 3 baculoviral genes and one of its DNA elements induces this reorganization in silkworm cells [20]. These genes, *ie1*, *lef3*, and *p143*, encode a transcriptional activator, single-stranded DNA-binding protein, and helicase, respectively. The DNA element *hr* can act as an enhancer and origin of replication [68]. The combined functions of these 4 factors indicate that the formation of a viral DNA synthesis complex is likely sufficient for baculoviruses to induce ROCC. Removing any of these 4 factors abrogates this ability during baculovirus infection [8].

Parallel findings from genetic analyses of EBV have shown that all 7 of its essential genes for productive or lytic DNA synthesis (*balf5*, *balf2*, *bblf2/3*, *bblf4*, *bslf1*, *bmlf1*, and *bmrf1*) and an origin specialized for lytic DNA synthesis (*oriLyt*) are required to induce chromatin reorganization [7]. Genetically knocking out one of these genes completely abrogates the reorganization, while using chemical inhibitors of viral DNA synthesis (thereby allowing only the formation of a DNA synthesis complex) supports the initial condensation of cellular chromatin without its margination (Fig 3). Completion of EBV’s productive phase leads to complete reorganization (ROCC type II), in which cellular chromatin is condensed and has moved to the periphery of the nucleus. At this stage, up to 30% of the DNA in the nucleus is chromatin-free, viral DNA [69]. The amplification of this viral DNA likely contributes mechanistically to type II reorganization. Thus, it seems that the initial chromatin condensation does not require more than the initiation of viral DNA synthesis, while the chromatin margination that follows requires extensive viral DNA synthesis. Proving that only the initiation of viral DNA synthesis suffices for the initial chromatin condensation is another uncertainty that will be important to resolve. This feature is a potential vulnerability of ROCC-inducing viruses.

## Being a DNA virus is insufficient to induce ROCC

While all the viruses that have been identified as capable of inducing ROCC are DNA viruses, not all DNA viruses induce ROCC. Two prominent properties shared by DNA viruses that induce ROCC are viral DNA replication within the host nucleus, and encapsidation of histone-free genomes within the virions. Accordingly, poxviruses, which replicate in the cytoplasm of their host cell, do not affect the distribution of chromatin in the nucleus [70]. Even within families of DNA viruses that replicate their genomes in the host nucleus, some do not induce this reorganization. For example, polyomaviruses and papillomaviruses replicate in the nucleus of infected cells and do not reorganize the host chromatin [71–73]. One difference that is likely pivotal is that these viruses encapsidate their genomes as histone-bound DNA [72–74], in contrast to ROCC-inducing viruses that encapsidate histone-free DNA.

SV40, a member of the polyomavirus family, was tested for its possible complementation for EBV’s *oriLyt*-mediated DNA amplification in promoting the reorganization of chromatin. EBV-triggered chromatin reorganization and the replication of SV40 DNA proved to be incompatible [7]. SV40 can replicate its DNA in cells that carry EBV, but not in cells in which ROCC has been induced. The lytic phase of EBV inhibits cellular DNA synthesis [23,75]; thus, the failure of cellular DNA synthesis is likely to affect SV40 through a shared mechanism involving the inhibition of histone-associated DNA synthesis. By contrast, virus-induced ROCC occurs concurrently with the histone-free DNA synthesis that takes place when EBV replicates its DNA productively, a feat that may well extend to other families of viruses that induce ROCC.

## Potential mechanisms mediating virus-induced ROCC

Using information gained from the properties shared by the viruses that induce ROCC (Table 1), and an appreciation of the viral genes it requires [7,20], we can formulate some possible mechanisms for virus-induced ROCC. In this formulation, we assume that the 5 families of viruses that induce chromatin reorganization use common mechanisms.

Geminiviruses and parvoviruses, which encode only a few genes, can induce a reorganization of their hosts’ chromatin. Their small coding capacity, spanning 2.7 to 5.2 kb, indicates that they cannot encode all the machinery needed to condense chromatin into chromatid-like structures [76]. Therefore, these viruses need to use cellular machinery to mediate this reorganization. One property that all ROCC-inducing viruses share is that their encapsidated DNA lacks cellular histones, making it likely that their DNA is synthesized without a requirement for depositing histones on it. In uninfected cells, inhibiting histone synthesis, with its concomitant decrease in the deposition of histones on newly synthesized DNA, inhibits fork progression [77]. Inhibiting the deposition of histones also induces cell death in uninfected cells [78]. Herpesviruses avoid this histone dependence. For example, in cells productively infected with HCMV, the virus specifically inhibits the synthesis of replication-dependent histones [52] while amplifying its DNA. In cells induced to support the lytic cycle of EBV, the virus inhibits chaperones associated with replication-dependent histones to block histone deposition while amplifying its DNA [23]. These shared properties support the notion that host cells recognize the atypical, newly synthesized, histone-free viral DNA and trigger the initial DNA condensation of ROCC type I (Fig 3). This condensation also occurs with geminiviruses and herpesviruses [7,19,23] and is consistent with the finding that blocking the initiation of viral DNA synthesis by using viral mutants null for a viral DNA polymerase blocks the triggering and the initial condensation of cellular DNA [7].

This initial chromatin condensation is followed by the movement of cellular chromatin to the nuclear margins for all 5 families of ROCC-inducing viruses. This movement could be mediated by the expansion of the sites of viral DNA amplification, as suggested for baculoviruses [8]. The sites of viral DNA amplification, which are often termed replication compartments (RCs), are not membrane bound, and, for parvoviruses, are discrete entities [79]. How the expansion of RCs (which occurs as the viral DNA is amplified in them) might mechanistically mediate the condensation of host chromatin is unknown. Elucidating this mechanism is an outstanding goal in the study of ROCC.

One possible mechanism that could underlie the movement of the chromatin during ROCC type II (Fig 3) depends on liquid–liquid phase separation (LLPS). Phase separation of liquids occurs when “there are two different compositions of all molecular species with the same chemical potentials in both phases” [80]. The high local concentrations of viral proteins and nucleic acids may form liquid phases separated from other liquid regions of the nucleus, as occurs for nucleoli [81]. In normal, uninfected cells, cellular chromatin likely exists in its own phase [82], and mitotic chromosomes clearly display LLPS [83], making LLPS a plausible mechanism to mediate virus-induced ROCC. All viruses that elicit ROCC encode some replication proteins that bind the viral genomes. Given that LLPS fosters the assembly of complexes that initiate DNA synthesis in *Drosophila* [84], a testable hypothesis is that the replication proteins and DNA of these viruses form a liquid phase distinct from that of condensing chromatin.

Recent studies using HCMV support a model in which LLPS contributes to the formation of ROCC type II [85]. In these studies, the UL112-113 protein products of HCMV were shown to form a liquid-like biomolecular condensate under multiple conditions, including during HCMV infection, following transfection of UL112-113 into cells, and in vitro, using purified UL112-113 proteins. The UL112-113 condensates are regions in which the polymerase processivity factor UL44 accumulates and viral DNA replication occurs. In addition, the formation and maintenance of these UL112-113 condensates is disrupted by treatment with 1,6-hexanediol, a solvent for LLPS. These findings support a model of ROCC type II in which, in this example, UL112-113 mediates the formation of RCs through LLPS and possibly recruits to them several essential replication proteins through the same mechanism [85]. The same study also included an examination of UL112-113–positive RCs using fluorescence recovery after photobleaching (FRAP), both early and later in infection as the compartments expanded. The early RCs recovered rapidly and completely from the photobleaching, while the mature compartments recovered slowly and only partially. Liquid-like condensates can become less fluid when there is an increased concentration of their constituents, particularly those that can act as scaffolds, such as DNA and RNA [86]. Recent observations [85] therefore support a model for ROCC type II in which the early RCs form through LLPS and become more gel-like as they mature and the amount of replicated DNA they contain increases.

The gel-like nature of the RCs of HCMV is shared by those of parvoviruses. FRAP analyses of cells infected by canine parvovirus have shown the diffusion of PCNA (a processivity factor for pol δ) in RCs to be more than an order of magnitude slower than in the nuclei of uninfected cells [10]. Furthermore, the finding that the inhibitor of viral DNA synthesis, phosphonoacetic acid (PAA), does not disrupt the formation of UL112-113–positive, early compartments but does prevent their maturation into more gel-like condensates [85] is consistent with the two-step model proposed for EBV-induced ROCC [7]. Using PAA to inhibit viral DNA amplification in EBV also results in ROCC type I [7], indicating that extensive viral DNA amplification is required to yield ROCC type II, possibly through LLPS.

Other recent studies are helping to explain how amplifying viral DNA can move cellular chromatin to the periphery of the nucleus in ROCC type II. Both chromatin and mitotic chromosomes behave as biomolecular, liquid-like condensates [82,83]. Tubulin, which is negatively charged, moves the condensates containing fragmented chromosomes around in cells as it polymerizes [83]. The negatively charged, amplifying viral DNA could provide a force, similar to that of polymerizing tubulin, driving the chromatin condensates to the nuclear boundaries.

While a role for LLPS in mediating the ROCC type II is appealing, it is not established. An examination of RCs formed by herpes simplex virus type I concluded that these compartments do not arise solely from LLPS but occur from the amplifying viral DNA being largely free of histones and serving as an attractive reservoir for DNA-binding proteins such as RNA polymerase II [87]. What is clear is that a role for expanding RCs in driving ROCC type II remains appealing based on parsimony: All viruses that elicit ROCC also form RCs separated from the marginated cellular chromatin.

## Why might viruses trigger chromatin reorganization?

A recently proposed megataxonomy of viruses indicates that virus-induced chromatin reorganization is likely to have arisen more than once in the evolution of the 5 families of ROCC-inducing viruses [88]. Viruses are polyphyletic, meaning that many virus groups do not share a common ancestry. In the proposed megataxonomy, the 5 families of viruses that induce ROCC largely belong to different realms, with Baculoviridae unable to be assigned into any realm, and Parvoviridae and Geminiviridae sharing a realm and kingdom [88]. As virus realms are not thought to share a common ancestry, it is likely that virus-induced ROCC arose separately in most of these 5 virus families. This convergent evolution underscores the importance of ROCC to the life cycles of the viruses that induce it.

How do the many viruses that induce this striking reorganization of their host’s chromatin benefit from it? All express essential, early viral genes from chromatin-bound templates at the beginning of their productive infections. Members of these 5 families also regulate modifications of cellular histones [89–93], presumably to optimize this early transcription. The transcription of viral structural genes for adenoviruses and herpesviruses occurs as viral DNA is amplified and likely uses templates free of nucleosomes [43,87,94]. For both EBV-infected and Kaposi’s sarcoma herpesvirus (KSHV)-infected cells, late viral transcription occurs on templates localized away from regions of condensed cellular chromatin [94,95]. By separating these viral transcription templates from cellular chromatin, ROCC likely favors viral RNA synthesis.

Another potential benefit of reorganizing cellular chromatin comes from condensed and inaccessible chromatin being associated with a repression of gene expression. The nuclear periphery is typically a repressive environment, in which case the virus-induced ROCC phenotype could accentuate the repression of transcription from compacted chromatin, as found in lamina-associated domains (LADs) [96,97]. Stretches of cellular DNA that associate with lamin B1, a constituent of the nuclear lamina, have been mapped and extend from 0.1 to 10 megabases in length [98] and, when summed over multiple cell types, can accommodate a third of all cellular chromatin. Most genes within these LADs are inhibited transcriptionally [99,100]. Type II ROCC leads to the cellular chromatin being condensed to the nuclear periphery, adjacent to the nuclear lamina [7,15]. It is likely that this localization leads to an increase in repression of cellular transcription, favoring the transcription of viral genes, thus contributing to virus-mediated host shutoff [101]. An analysis of both cellular and viral transcription during the early phase of the lytic cycle of EBV supports this conclusion; the expression of 99% of cellular genes on average was inhibited 3-fold, while that of some viral genes was increased 100-fold or more [102]. Local infection of tobacco leaves by the geminivirus tomato yellow leaf curl virus also affects cellular gene expression, but less substantially, inhibiting only 64% of detected, differentially expressed genes [103]. Chromatin reorganization, therefore, could benefit the viruses that induce it by suppressing host gene expression during production of progeny viruses.

## What can be gained by understanding virus-induced ROCC?

Many viruses that damage human health or our ecosystem also induce chromatin reorganization: Geminiviruses cause billions of dollars in crop loss of beans, tomatoes, and cassava production annually [104–106]; parvoviruses cause severe diseases in dogs and cats and also are a major cause of infertility in pigs [107]; adenoviruses can cause respiratory illnesses in humans and infect a variety of canines, causing, for example, fatal infections in red foxes [108]; baculoviruses were feared for their threat to silkworms but have proven beneficial in North America in controlling infestations of gypsy moths, which are destructive to hardwood trees [33]; and herpesviruses cause many diseases in humans, ranging from chicken pox to cancers [109]. Chromatin reorganization may therefore be a vulnerability shared by these viruses that we can exploit to limit their pathogenesis.

Unfortunately, ROCC type II, in which viral DNA synthesis expands to fill the centers of infected nuclei as the cellular chromatin is moved to the periphery of the nuclei, may be an intractable target. These viruses all depend on some, but different, host cell functions to synthesize their DNA. Inhibiting type II ROCC will likely require targeting cellular functions that are also needed by uninfected cells, therefore making it deleterious to the host. Conversely, ROCC type I, in which the chromosomal DNA is initially condensed, is an attractive target. Both geminiviruses and herpesviruses display this type I phenotype. As these viruses affect a wide range of plants and vertebrates and have genomes that differ in size by 100-fold, it is likely that all 5 families also induce ROCC through a similar, two-step progression. The available data indicate that ROCC type I is a cellular response, in which the infected cells recognize newly synthesized, histone-free viral DNA, which then triggers the initial cellular chromatin condensation. Characterizing this initial triggering mechanism may allow us to target virus-infected cells without compromising uninfected cells within the host, thus helping us to limit the pathogenicity of these viruses.

## Conclusions

DNA viruses often kill infected cells after they produce their progeny. This killing is an ultimate takeover. However, viruses need also to manipulate their hosts so that as many progeny as possible can be produced prior to cell death. Five families of viruses orchestrate an unexpected reorganization of their host cells’ chromatin as they amplify their own DNA. By doing so, they spatially separate viral, transcribed DNA from that of the host, segregating the host’s chromatin to peripheral sites that are more likely to be transcriptionally silenced. This segregation is one possible strategy to shutoff the host. Virus-induced chromatin reorganization also spatially separates the histone-free viral DNA to be encapsidated from the histone-bound cellular DNA. How they accomplish this multipronged takeover remains uncertain, but the recently appreciated LLPS is a candidate for mediating the movement of the cellular chromatin to the nuclear periphery. This dramatic takeover of the host nucleus by so many DNA viruses is fascinating, a rich source for new insights into how they so successfully dominate their hosts, and a potential point for intervening therapeutically in their life cycles. Although much remains uncertain about virus-induced ROCC, addressing outstanding questions such as whether ROCC always occurs in 2 stages and how the initial chromatin condensation is triggered will help to move these initial observations towards real-world applications.

## Abbreviations

EBV: Epstein–Barr virus
FISH: fluorescence in situ hybridization
FRAP: fluorescence recovery after photobleaching
HBoV1: human bocavirus 1
HCMV: human cytomegalovirus
KSHV: Kaposi’s sarcoma herpesvirus
LAD: lamina-associated domain
LLPS: liquid–liquid phase separation
MVM: minute virus of mice
PAA: phosphonoacetic acid
RC: replication compartment
ROCC: reorganization of cellular chromatin
